# Comment on ‘Effect of anatomical liver resection for hepatocellular carcinoma: a systematic review and meta-analysis’

**DOI:** 10.1097/JS9.0000000000001154

**Published:** 2024-02-13

**Authors:** Jia-Xi Mao, Han-Xiang Zhong, Xin-Yi Lu, Yuan-Yu Zhao, Li-Ye Zhu, Hong Fu, Guo-Shan Ding, Fei Teng, Wen-Yuan Guo

**Affiliations:** a Department of Liver Surgery and Organ Transplantation, Changzheng Hospital; b Department of Immunology and Medical Immunology State Key Laboratory, Naval Medical University, Shanghai, People’s Republic of China

*Dear Editor*,

We were highly interested in the systematic review and meta-analysis conducted by Shin *et al*.^1^, which examined the efficacy of hepatic anatomical resection (AR) for hepatocellular carcinoma (HCC). The study included 22 propensity score matching (PSM) studies and compared the outcomes of AR versus nonanatomical resection (NAR). The results showed that AR was associated with better overall survival (OS) and recurrence-free survival (RFS), as well as lower rates of local and multiple intrahepatic recurrences, particularly for HCC without liver cirrhosis and with a tumor diameter of ≤5 cm. This systematic review and meta-analysis has strong clinical utility and addresses an issue of great interest to frontline clinicians, providing valuable insights for clinical decision-making. However, there are still some points for consideration and improvement during the writing process.

Firstly, considering that the Milan criteria are superior to tumor size alone in the assessment of early-stage HCC, it would be helpful to include the use of Milan criteria instead of a tumor diameter of ≤5 cm in the subgroup analysis. This could increase the number of eligible studies for analysis and improve accuracy.

Secondly, some of the included studies were sourced from the same clinical centers with overlapping time periods, which may result in data duplication and affect the credibility of the analysis. For example, Famularo *et al*. published two articles based on data from HCC patients at two institutions (Grande Ospedale Metropolitano Niguarda and San Gerardo Hospital)^2,3^. The time periods covered by these two articles show overlap, with January 2001 to August 2015^2^ and January 2005 to January 2016^3^. This leads to the statistical redundancy and offset in the forest plots presented in Figure 3^1^ and 5^1^. Additionally, Zhao *et al*.^4^ published two articles in 2017 and 2020^5^, respectively, both from the same clinical center (the Affiliated Wuxi No. 2 People’s Hospital of Nanjing Medical University) but with different time periods, from January 2004 to December 2013^4^ and January 2010 to December 2015^5^. As there is partial overlap in the time periods, these two articles should not be simultaneously included in the forest plots presented in Figure 2^1^ and 3^1^. Similarly, Kaibori *et al*.^6^ published two correlated articles in 2017 and 2020^7^, conducted within the same time period and with the same ethics committee number. The latter^7^ is a subset of the former^6^, so they should not be simultaneously included in the forest plots presented in Figures 2^1^, 3^1^, and 6^1^. Furthermore, there is a minor error that needs to be rectified: The year label for Kaibori (Japan, Korea) in Table 1^1^ should be 2017 instead of 2020.

Last but not the least, several recent publications have compared the efficacy of AR versus NAR for HCC using PSM. Kwon *et al*.^8^ found that after PSM, when the tumor size was <5 cm, AR group (*N*=224) had better OS and RFS rates than NAR group (*N*=117). However, when the tumor size was ≥5 cm, there was no significant difference in OS and RFS rates between AR group (*N*=148) and NAR group (*N*=51). Tang *et al*.^9^ reported a multicenter study analyzing the efficacy of AR for early-stage HCC (BCLC stage 0/A) using PSM, which once again confirmed that AR can reduce tumor recurrence risk and improve OS and RFS in early-stage HCC patients. For the initial treatment of early-stage HCC, as long as the surgical technique is feasible, AR is recommended as the first choice. Lee *et al*.^10^ compared laparoscopic anatomical resection (LAR) with laparoscopic nonanatomical resection (LNAR) for posterior segment (PS) HCC using PSM and found that LNAR is a safe and feasible procedure with comparable oncological outcomes to LAR. Therefore, LNAR can be considered when the tumor is located in the PS and LAR cannot be performed. These publications provide valuable insights and should be considered for inclusion in future updates of this systematic review and meta-analysis.

We sincerely appreciate the contributions made by the authors to this research, which have provided clinicians with a deeper understanding and knowledge of the efficacy of AR for HCC, thereby advancing the field of hepatic AR. We look forward to the authors taking these mentioned points into consideration to enhance the rigor and reliability of their study findings.

## Ethical approval

Not applicable.

## Consent

Not applicable.

## Sources of funding

This research was supported by the National Natural Science Foundation of China (82371792, 81971503), Open Research Project Program of the State Key Laboratory of Medical Immunology (NKLMI2023K03), Basic medical research project of Naval Medical University (2022QN072), and Clinical technology innovation project of Shanghai Shenkang Hospital Development Center (SHDC12020104).

## Author contribution

J.X.M.: conceptualization and writing – original draft; H.X.Z.: formal analysis and writing – original draft; X.Y.L.: formal analysis and writing – original draft; Y.Y.Z.: investigation and resources; L.Y.Z.: investigation and resources; H.F.: supervision; G.S.D.: supervision; F.T.: funding acquisition and writing – review and editing; W.Y.G: funding acquisition, project administration, and supervision.

## Conflicts of interest disclosure

The author declares no conflict of interest.

## Research registration unique identifying number (UIN)

Not applicable.

## Guarantor

Wen-Yuan Guo, e-mail: guowenyuan@smmu.edu.cn; Fei Teng, e-mail: tengfei@smmu.edu.cn.

## Data availability statement

Not applicable.

## Provenance and peer review

Not applicable.
